# A Secondary Antibody-Detecting Molecular Weight Marker with Mouse and Rabbit IgG Fc Linear Epitopes for Western Blot Analysis

**DOI:** 10.1371/journal.pone.0160418

**Published:** 2016-08-05

**Authors:** Wen-Wei Lin, I-Ju Chen, Ta-Chun Cheng, Yi-Ching Tung, Pei-Yu Chu, Chih-Hung Chuang, Yuan-Chin Hsieh, Chien-Chiao Huang, Yeng-Tseng Wang, Chien-Han Kao, Steve R. Roffler, Tian-Lu Cheng

**Affiliations:** 1 Institute of Biomedical Sciences, National Sun Yat-Sen University, Kaohsiung, Taiwan; 2 Graduate Institute of Medicine, Kaohsiung Medical University, Kaohsiung, Taiwan; 3 Center for Biomarkers and Biotech Drugs, Kaohsiung Medical University, Kaohsiung, Taiwan; 4 Department of Public Health and Environmental Medicine, College of Medicine, Kaohsiung Medical University, Kaohsiung, Taiwan; 5 Department of Medical Laboratory Science and Biotechnology, College of Health Sciences, Kaohsiung Medical University, Kaohsiung, Taiwan; 6 Graduate Institute of Clinical Medicine, Kaohsiung Medical University, Kaohsiung, Taiwan; 7 Department of Biomedical Science and Environmental Biology, Kaohsiung Medical University, Kaohsiung, Taiwan; 8 Department of Biochemistry, Kaohsiung Medical University, Kaohsiung, Taiwan; 9 Institute of Biomedical Sciences, Academia Sinica, Taipei, Taiwan; Duke University Medical Center, UNITED STATES

## Abstract

Molecular weight markers that can tolerate denaturing conditions and be auto-detected by secondary antibodies offer great efficacy and convenience for Western Blotting. Here, we describe M&R LE protein markers which contain linear epitopes derived from the heavy chain constant regions of mouse and rabbit immunoglobulin G (IgG Fc LE). These markers can be directly recognized and stained by a wide range of anti-mouse and anti-rabbit secondary antibodies. We selected three mouse (M1, M2 and M3) linear IgG1 and three rabbit (R1, R2 and R3) linear IgG heavy chain epitope candidates based on their respective crystal structures. Western blot analysis indicated that M2 and R2 linear epitopes are effectively recognized by anti-mouse and anti-rabbit secondary antibodies, respectively. We fused the M2 and R2 epitopes (M&R LE) and incorporated the polypeptide in a range of 15–120 kDa auto-detecting markers (M&R LE protein marker). The M&R LE protein marker can be auto-detected by anti-mouse and anti-rabbit IgG secondary antibodies in standard immunoblots. Linear regression analysis of the M&R LE protein marker plotted as gel mobility versus the log of the marker molecular weights revealed good linearity with a correlation coefficient R^2^ value of 0.9965, indicating that the M&R LE protein marker displays high accuracy for determining protein molecular weights. This accurate, regular and auto-detected M&R LE protein marker may provide a simple, efficient and economical tool for protein analysis.

## Introduction

Molecular weight markers that can be auto-detected by secondary antibodies and tolerate denaturing conditions can offer great convenience for protein analysis by Western Blotting. Currently, dye-conjugated molecular weight protein markers are commonly used in protein analysis [1, 2]. However, the conjugation of dye molecules to protein markers may altered their electromobility and impede precise molecular weight determinations [3]. To overcome this problem, a number of dye-free and automatically color developed molecular weight markers have been developed by fusion of the functional domains of specific proteins. For example, Chang and colleagues developed a green fluorescent protein (GFP)-based protein ladder which can be monitored on SDS-PAGE gel under ultraviolet (UV) illumination [4]. Bischof and colleagues developed a heme-based ladder which possess peroxidase activity that can be revealed by substrate treatment [5]. Immunoglobulin (Ig)-binding domain fusion proteins have been developed as auto-color-development molecular weight markers, such as the EasySee Western marker (Spark Biologicals Technology) which contains IgG-binding domains of protein A and protein G [6]. Unfortunately, a limitation of this class of markers is that they must be used under non-denaturing conditions, which may lead to inaccurate determination of molecular weight [7]. Although several boilable marker products have been developed, such as hexahistidine-tagged [8] and S-tagged protein ladders [9] (Perfect Protein Western Markers, Novagen) and the Mega-tag ladder [7], these markers require the use of tag-specific primary antibodies and are not compatible for measurement of tag-free proteins. Thus, development of an auto-detected, denaturable, precise and widely applicable molecular weight marker is desirable.

**Fig 1:**
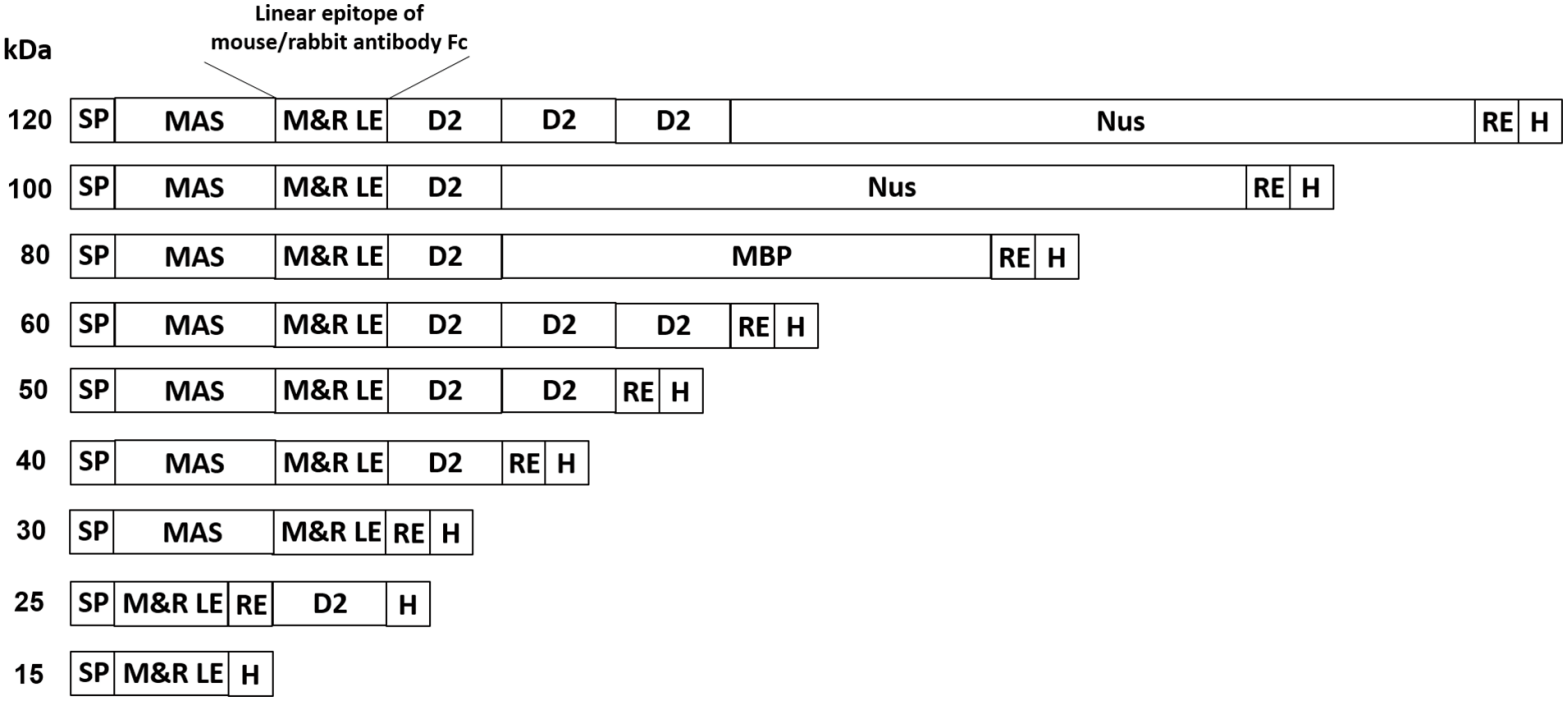
Construction of M&R LE protein marker for Western blotting. We fused mouse and rabbit linear eptitopes (M&R LE) to generate a 15 kDa leader peptide which can be recognized by anti-mouse or anti-rabbit IgG secondary antibodies. The M&R LE molecular weight ladder was constructed by fusing a leader peptide with the *E*. *coli* outer membrane protein intimin domain 2 (D2), maltose-binding protein (MBP) or *E*. *coli* transcription factor (Nus) polypeptides. The M&R LE protein marker can be directly visualized on Western blotting by staining with anti-mouse or anti-rabbit IgG secondary antibodies. SP, signal peptide; M2R2, M&R epitope; RE, restriction enzyme sites; MAS, MW adjusting sequence; H, 6X His tag.

To develop an accurate, denaturable and auto-detecting molecular weight marker, we selected three mouse (M1, M2, M3) and three rabbit (R1, R2, R3) linear epitopes (LE) through prediction of the crystal structures of mouse IgG1 and rabbit IgG heavy chain constant regions by ABC prep, Bepi prep and C.V.C. software. We expected that the linear epitopes exposure at constant region of primary antibodies are potentially bound by secondary antibodies after structural destroyed by heating [10–17]. We determined the ability of secondary antibodies to directly detect these linear epitopes by Western blotting. We fused the best mouse and rabbit linear epitopes (M&R LE) and fused the resulting 15 kDa peptide to the *E*.*coli* outer membrane protein intimin domain 2 (D2, 10 kDa), maltose-binding protein (MBP, 40 kDa) or an *E*.*coli* transcription factor (Nus, 60 kDa) to generate 15–120 kDa molecular weight marker proteins (M&R LE protein marker) (Fig 1). The production of M&R LE protein markers in *E*. *coli* (BL21) was confirmed by anti-His staining and Western blotting. The auto-detecting nature of the M&R LE protein markers was examined by direct staining on immunoblots with anti-mouse and anti-rabbit secondary antibodies. The molecular weight accuracy of the M&R LE protein marker was also determined by calculating the correlation (R^2^ value) of the relative mobilities versus the logarithmic molecular weights in comparison with a commercial prestained marker. The M&R LE protein marker can be directly recognized by secondary antibodies under denaturing conditions for automatic visualization on immunoblots. These new markers provide an accurate, convenient and effective tool for protein analysis and proteomic research.

## Materials and Methods

### Antibodies

Secondary antibodies that were used to directly stain immunoblots included: horseradish peroxidase (HRP)-labeled goat anti-mouse IgG Fc (115-035-006), HRP-goat anti-rabbit IgG (115-035-003), HRP-goat anti-mouse IgG, A, M (115-035-006), HRP-goat anti-mouse IgM (109-035-006) (Jackson Immuno-Research Laboratories, Westgrove, PA, USA), HRP-goat anti-mouse IgG (H+L) (626520) (Invitrogen, Carlsbad, CA, USA), HRP-goat anti-mouse IgG (H+L) (31430) and HRP-goat anti-rabbit IgG (H+L) (31460) (Thermo Scientific, Waltham, MA, USA), and HRP-goat anti-rabbit IgG (H+L) (656120) (Invitrogen, Carlsbad, CA, USA). All secondary antibodies were diluted 1000-fold in phosphate buffered saline (PBS) with 5% milk for immunoblotting experiments.

### Computational Prediction of Linear Epitopes in the Fc Region of Mouse and Rabbit Antibodies

The gene sequences of mouse IgG1 (PDB number: 3HKF) and rabbit IgG antibodies (PDB number: 2VUO) were obtained from the National Center of Biotechnology Information (NCBI) and the Protein Data Bank (PDB). ABCpred (http://www.imtech.res.in/raghava/abcpred/), BepiPred 1.0 Server (http://www.cbs.dtu.dk/services/BepiPred/) and Cancer Vaccine Center (CVC) bioinformatics (http://bio.dfci.harvard.edu/research/bioinformatics-tools.php) were used to predict the linear epitopes in the antibody Fc regions. Three mouse (M1, M2 and M3) and three rabbit (R1, R2 and R3) linear epitope candidates were selected based on the predicted results. The M1 (TPKVTCVVVDISKDDPEVQF SWFVDDVEVHTAQTQPREEQFNSTFRSVSELP), M2 (EFKCRVNSAA FPAPIEKTISKTKGRPKAPQVYTIPPPKEQMAKDKVSLTCMITD) and M3 (QPIMNTNGSYFVYSKLNVQKSNWEAGNTFTCSVLHEGLHNHHTEKSLSHSPGL) peptides were derived from the mouse IgG1 heavy chain CH_2_ and CH_3_ domains. The R1 (PKDTLMISRTPEVTCVVVDVSQDDPEVQFTWYINNEQVRT ARPPLREQQFNSTIRVVSTLPIT), R2 (GKEFKCKVHNKALPAPIEKT ISKARGQPLEPKVYTMGPPREELSSRSVSLTCMINGFYPSDISVEW) and R3 (KNGKAEDNYKTTPAVLDSDGSYFLYNKLSVPTSEWQRGDVFTCSVMHEANHYTQKSISRSPGK) peptides were derived from the rabbit IgG heavy chain CH_2_ and CH_3_ domains.

### Construction of M&R LE Protein Marker

The polymerase chain reaction (PCR) was used to amplify genes including molecular adjusting sequence (MAS; 15 kDa), O157 *Escherichia coli* (*E*.*coli*) outer membrane protein intimin domain 2 (D2; 10 kDa), maltose-binding protein (MBP; 40 kDa) and an *E*.*coli* transcription factor (Nus; 60 kDa). Candidate DNA fragments of M1, M2, M3, R1, R2 and R3 peptides were assembled by PCR and cloned into the pRSETB plasmid (Invitrogen). The M2R2 fusion sequences were created by digesting the R2 sequence with BglII and PciI and subcloning the DNA fragment into the pRSETB-M2 plasmids to generate pRSETB-M2R2 plasmids (M&R epitope). A sequence coding for a His-tag (six histidine) was digested with NcoI and AvaI and inserted behind the M2R2 fusion sequence to generate pRSETB-M2R2-His tag (15 kDa). The pRSETB-M2R2-His tag (15 kDa) was digested with AvaI and the D2 DNA fragment was inserted to generate pRSETB-M2R2-D2-His tag (25 kDa). pRSETB-M2R2-His tag (15 kDa) was digested with NdeI and EcoRI and the MAS DNA fragment was inserted in front of the M2R2 sequence to generate pRSETB-MAS-M2R2-His tag (30 kDa). pRSETB-MAS-M2R2-His tag (30 kDa) was further digested with AvaI and ClaI and the D2 DNA fragment was inserted to create pRSETB-MAS-M2R2-D2-His tag (40 kDa). pRSETB-MAS-M2R2-D2-His tag (40 kDa) was digested with KpnI and EcoRI and another D2 DNA fragment was inserted to create pRSETB-MAS-M2R2-D2-D2-His tag (50 kDa). Consequently, pRSETB-MAS-M2R2-D2-D2-D2-His tag (60 kDa) was generated by PmlI and KpnI digestion of pRSETB-MAS-M2R2-D2-D2-His tag (50 kDa) and insertion of another D2 gene sequence. To construct pRSETB-MAS-M2R2-D2-MBP-His tag (80 kDa), pRSETB-MAS-M2R2-D2-His tag (40 kDa) was digested with ClaI and StuI, and then a fragment coding MBP was inserted behind the D2 sequence. pRSETB-MAS-M2R2-D2-Nus-His tag (100 kDa) was created by HindIII and EcoRI digestion of pRSETB-MAS-M2R2-D2-His tag (40 kDa) and insertion of a fragment coding the Nus sequence. Finally, the pRSETB-MAS-M2R2-D2-D2-D2-Nus-His tag (120 kDa) was generated by HindIII and ClaI digestion of pRSETB-MAS-M2R2-D2-D2-D2-His tag (60 kDa) and insertion of a Nus sequence.

### Expression of M&R LE Protein Marker

The plasmids of the M&R LE marker proteins were expressed in *E*. *coli* BL21 (DE3) (Invitrogen) cultured in Luria bertani (LB) broth containing 0.1 mg/mL ampicillin (Calbiochem, Merck KGaA, Darmstadt, Germany) at 37°C. When the culture absorbance at 600 nm reached 0.5, protein expression was induced by adding 0.2 mM isopropyl β-D-thiogalactopyranoside (IPTG) (Sigma-Aldrich, St. Louis, MO, USA) at 37°C for 3 hours. The transformed BL21 cells were collected for Western blotting analysis. The components of the M&R LE protein marker were stored in the refrigerator at -20°C.

### Western Blot Analysis of M&R LE Protein Marker

5 × 10^6^ BL21 cells expressing M&R LE protein markers were harvested, mixed with sodium dodecyl sulfate (SDS) loading dye containing β-mercaptoethanol (Sigma-Aldrich) and boiled for 10 min. Five to twenty μL samples were electrophoresed on a 10% or 12.5% SDS-PAGE and then electrophoretically transferred to nitrocellulose (NC) membranes (Millipore, Billerica, MA, USA). The membranes were blocked in PBS containing 5% milk at room temperature for 2 hours. For analysis of protein expression, the NC membranes were stained with mouse anti-histidine (His) tag antibody (AbD Serotec, UK) at room temperature for 1 hour. After washing 3-times with PBS containing 0.1% Tween-20 (Sigma), the NC membranes were incubated with goat anti-mouse IgG Fc-HRP (Jackson Immuno-Research Laboratories) at room temperature for 1 hour. After extensive washing, the blots were developed by ECL (Millipore, Billerica, MA, USA) and chemiluminescence was captured on a UVP BioImaging system (UVP, Cambridge, UK). To test the auto-detected activity of the M&R LE protein marker, the marker proteins were transferred onto NC membranes and directly stained with goat anti-mouse IgG Fc-HRP, goat anti-rabbit IgG Fc-HRP (Jackson Immuno-Research Laboratories), goat anti-mouse IgG (H+L)-HRP (Invitrogen) or goat anti-rabbit IgG (H+L)-HRP (Thermo Scientific) secondary antibodies at room temperature for 1 hour and then detection was performed as described above.

### Relationship between Molecular Weight and Electrophoretic Mobility of M&R LE Protein Markers

To examine the relationship between molecular size and mobility of M&R LE marker proteins, the logarithm of the protein molecular weights (in kDa) was plotted against the relative mobility (Rf) of the M&R LE marker proteins on immunoblots. The data was fit by simple linear regression using SigmaPlot 10.0 software (SYSTAT software, Inc., Chicago, IL, USA).

## Results

### Generation of M&R Linear Epitope for Anti-Mouse/Rabbit Secondary Antibody Recognition

**Fig 2:**
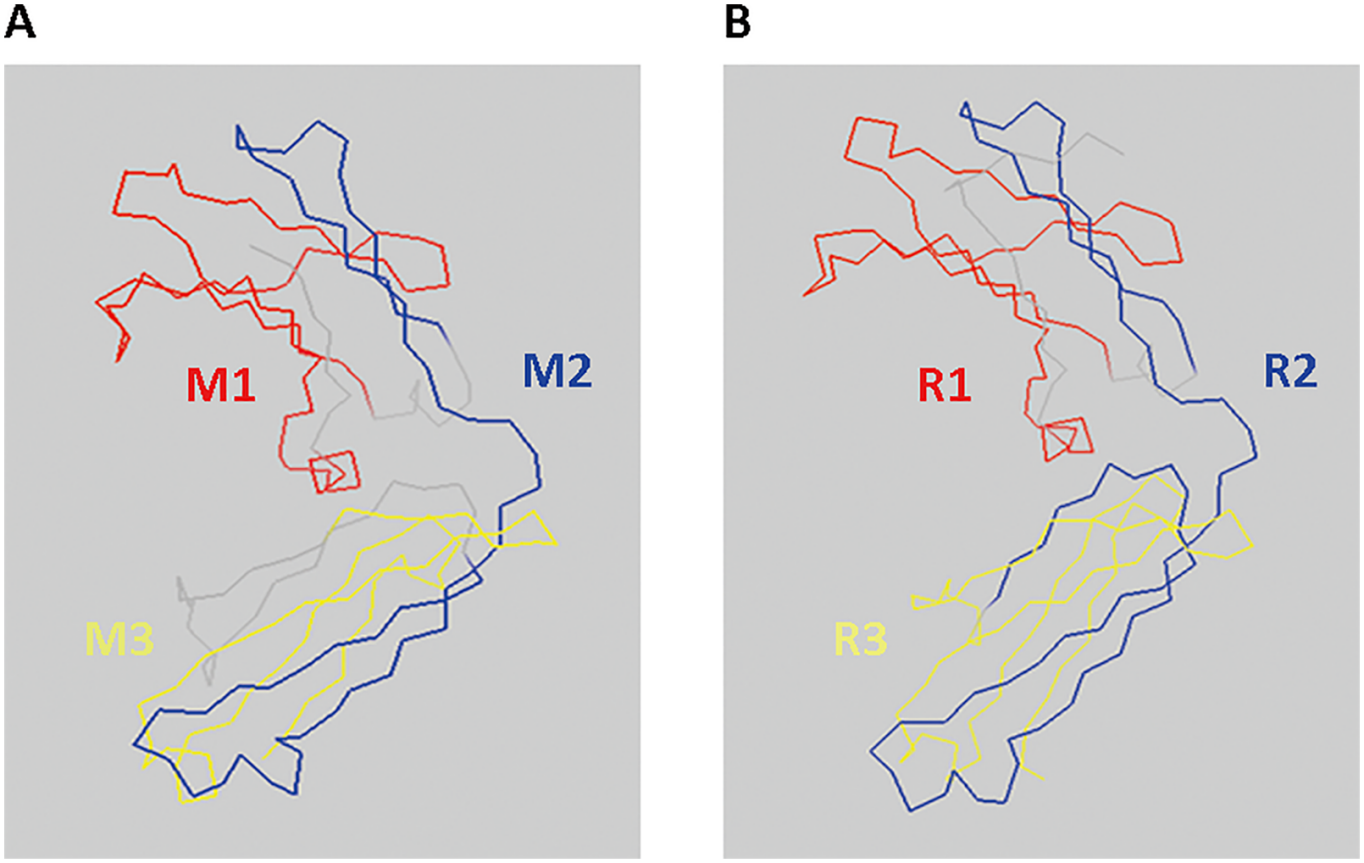
Prediction of linear epitope in mouse and rabbit antibody IgG Fc. To select linear epitopes, we predicted the crystal structures of mouse IgG1 and rabbit IgG Fc by ABCpred, BepiPred 1.0 Server and Cancer Vaccine Center (CVC) bioinformatics software. The 3D structures of mouse IgG1 and rabbit IgG Fc are displayed by PyMol software. (A) Three linear epitope candidates for mouse IgG1 Fc are shown as M1 (red), M2 (blue) and M3 (yellow). (B) Three linear epitope candidates for rabbit IgG Fc are shown as R1 (red), R2 (blue) and R3 (yellow).

**Fig 3:**
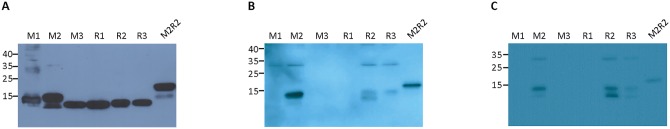
Expression and recognition of M&R LE leader peptides by mouse and rabbit secondary antibodies. The LE candidates (M1, M2, M3, R1, R2 and R3) and M&R LE leader peptide (M2R2) were separated by a 12.5% SDS-PAGE and transfered onto NC membrane. The expression of polypeptides was detected by staining with (A) anti-His tag antibody and standard Western Blotting. The recognition of LE candidates and M&R LE leader peptides on immunoblots by (B) HRP-conjugated anti-mouse or (C) HRP-conjugated anti-rabbit IgG Fc secondary antibodies. The chemiluminescence was captured by a UVP BioImaging system. The relative molecular weight (kDa) of commercial prestained markers is indicated on the left.

To develop a denaturable molecular weight marker that can be directly recognized by secondary antibodies, we designed marker proteins with linear epitopes derived from the Fc region of mouse IgG1 and rabbit IgG antibodies (IgG Fc LE) for binding by commercial secondary antibodies. IgG Fc LE candidate sequences from mouse (M1, M2 and M3) primary IgG1 and rabbit (R1, R2 and R3) IgG antibodies were predicted based on their crystal structures by ABCpred, BepiPred 1.0 Server and Cancer Vaccine Center (CVC) bioinformatics software (Table 1 and Fig 2). We cloned the IgG Fc LE candidate sequences into the bacterial expression vector pRSETB and expressed the peptides in BL-21 *E*. *coli*. Bacterial lysates were resolved on a SDS-PAGE and immunoblotted with an anti-His tag antibody to detect the His epitope tag present in the C-terminus of the IgG Fc LE. All six IgG Fc LE peptides were expressed in BL-21 *E*. *coli* (Fig 3A). To investigate whether these IgG Fc LE could be directly recognized by anti-immunoglobulin secondary antibodies, we stained the candidate LE peptides in immunoblots with horseradish peroxidase (HRP)-conjugated anti-mouse (Fig 3B) or anti-rabbit (Fig 3C) secondary antibodies. The M2 and R2 sequences appeared to be best recognized by HRP-conjugated anti-mouse secondary antibody (Fig 3B) and HRP-conjugated anti-rabbit secondary antibody (Fig 3C), respectively. We also observed that anti-mouse secondary antibody was slightly cross-reactive with R2 (Fig 3B). A similar result was observed for cross reactivity of anti-rabbit secondary antibody to M2 (Fig 3C). These data suggest that the M2 and R2 peptides are most strongly recognized by secondary antibodies.

**Table 1:** The amino acid sequences of linear epitope candidates of mouse IgG1 and rabbit IgG.

Linear epitope	Amino Acid Sequences	Molecular weight (kDa)
M1	TPKVTCVVVDISKDDPEVQFSWFVDDVEVHTAQTQPREEQFNSTFRSVSELP	5.94
M2	EFKCRVNSAAFPAPIEKTISKTKGRPKAPQVYTIPPPKEQMAKDKVSLTCMITD	5.94
M3	QPIMNTNGSYFVYSKLNVQKSNWEAGNTFTCSVLHEGLHNHHTEKSLSHSPGL	5.83
R1	PKDTLMISRTPEVTCVVVDVSQDDPEVQFTWYINNEQVRTARPPLREQQFNSTIRVVSTLPIT	7.26
R2	GKEFKCKVHNKALPAPIEKTISKARGQPLEPKVYTMGPPREELSSRSVSLTCMINGFYPSDISVEW	7.37
R3	KNGKAEDNYKTTPAVLDSDGSYFLYNKLSVPTSEWQRGDVFTCSVMHEALHNHYTQKSISRSPGK	7.33

We fused the M2 and R2 sequences by assembly PCR to generate an M&R epitope and expressed this combined epitope in BL-21 *E*. *coli*. Immunoblotting with anti-His tag antibody showed that the M&R epitope was successfully expressed by BL-21 *E*.*coli* (Fig 3A). The M2R2 M&R epitope was also recognized by both anti-mouse and anti-rabbit secondary antibodies in immunoblots (Fig 3B and 3C). We conclude that the M2R2 M&R epitope is suitable for construction of MW markers.

### Generation of M&R LE Protein Markers with Regular Molecular Weights and Auto-Detecting Ability for Western Blotting

**Fig 4:**
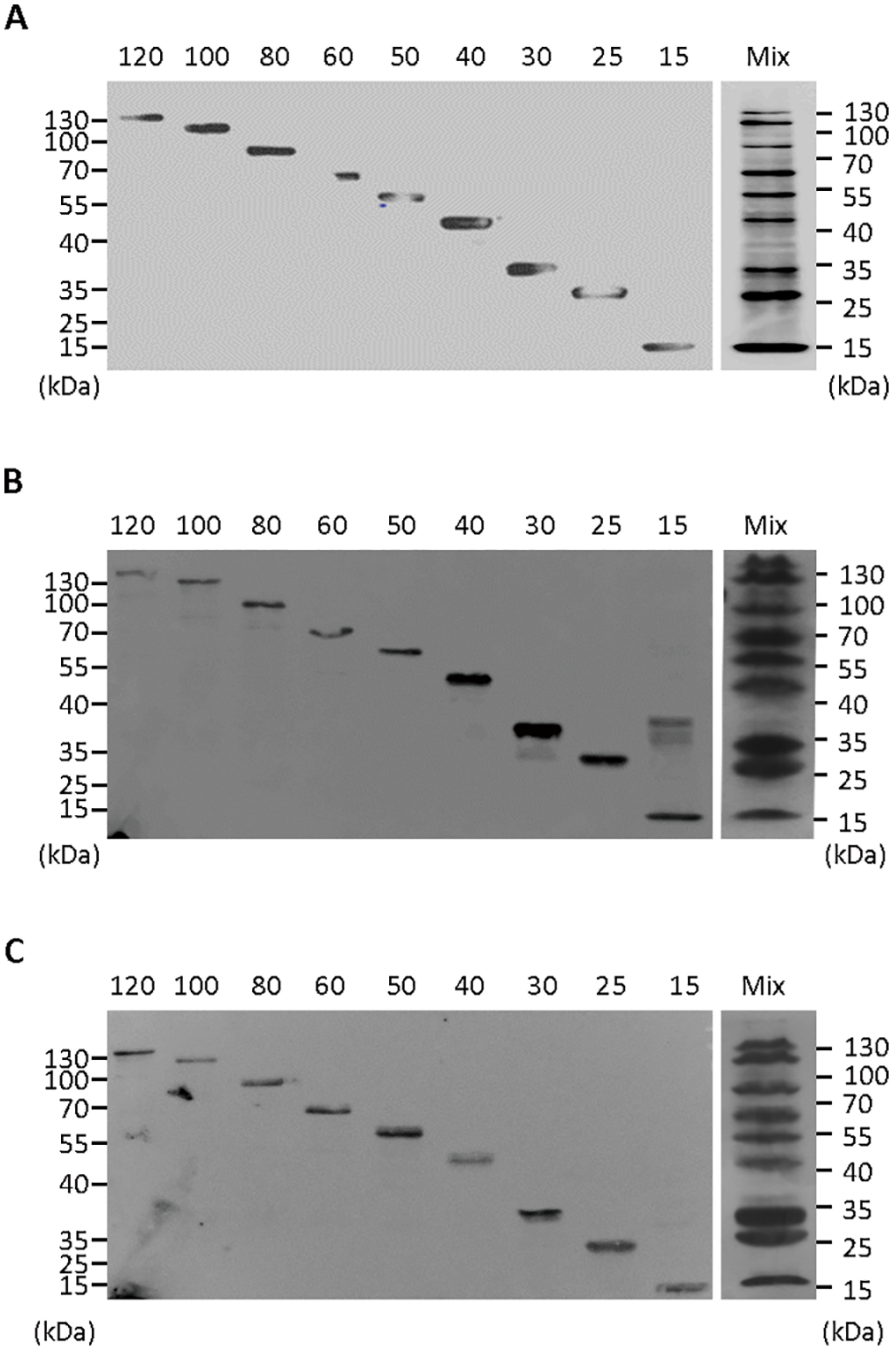
M&R LE protein marker with defined molecular weights. The M&R LE leader peptide (M2R2, 15 kDa) was assembled with D2, MBP or Nus to generate 25–120 kDa molecular weight marker proteins (M&R LE protein marker). (A) The expression of individual (left) or mixed (right) M&R marker proteins was determined by staining with anti-His tag secondary antibody. The binding of secondary antibodies to individual (left) or mixed (right) M&R marker proteins was determined by staining with HRP-conjugated (B) anti-mouse or (C) anti-rabbit IgG Fc secondary antibodies. The relative molecular weights (kDa) of a commercial prestained marker are indicated on the left and right. The chemiluminescence was captured by an UVP BioImaging system.

To generate a molecular weight ladder which can be directly recognized and auto-detected by HRP-conjugated anti-mouse or anti-rabbit IgG secondary antibodies, we fused the M&R epitope to recombinant proteins with different molecular weights (O157 *E*.*coli* outer membrane protein intimin domain 2 [D2; 10 kDa], maltose-binding protein [MBP; 40 kDa] or an *E*.*coli* transcription factor [Nus; 60 kDa]) to form a protein ladder with regular molecular weights (M&R LE protein marker, 15–120 kDa, Fig 1). All M&R LE protein markers could be expressed in BL-21 *E*.*coli* as determined by immunoblotting individual (Fig 4A, left) and mixed (Fig 4A, right) marker proteins with anti-His secondary antibody. Direct staining of individual (Fig 4B and 4C, left) and mixed (Fig 4B and 4C, right) marker proteins with HRP-conjugated anti-mouse or anti-rabbit IgG Fc secondary antibodies demonstrated a regular molecular weight ladder (15–120 kDa). Similar results were obtained using other HRP-conjugated anti-mouse or anti-rabbit secondary antibodies in immunoblots (Table 2). In addition, a mixture of M&R LE protein markers could also be visualized with HRP-conjugated anti-mouse or anti-rabbit IgG Fc secondary antibodies after separation in an 8% SDS-PAGE. However, there was lower resolution for low molecular weight marker proteins (15–30 kDa) (data not shown). To examine the sensitivity of our M&R LE protein marker, we separated two-fold serial dilutions of a mixture of the marker proteins on an SDS PAGE, transferred the markers to NC paper and then directly stained the membrane with secondary antibodies. S1 Fig shows that an 8-fold dilution of the M&R LE protein markers can be sensitively detected by anti-mouse (S1A Fig) or anti-rabbit (S1B Fig) secondary antibodies. We conclude that the M&R LE protein markers can be directly visualized by anti-mouse and anti-rabbit secondary antibodies in immunoblots.

**Table 2:** Commercial secondary antibodies tested for recognition of the M&R LE protein marker in Western Blots.

Secondary antibody	Auto-detected activity	Company (Catalog no.)
Goat anti-mouse IgG Fc-HRP	Yes	Jackson lab (115-035-008)
Goat anti-rabbit IgG Fc-HRP	Yes	Jackson lab (111-035-008)
Goat anti-mouse IgG (H+L)-HRP	Yes	Invitrogen (626520)
Goat anti-mouse IgG (H+L)-HRP	Yes	Thermo (31430)
Goat anti-rabbit IgG (H+L)-HRP	Yes	Invitrogen (656120)
Goat anti-rabbit IgG (H+L)-HRP	Yes	Thermo (31460)
Goat anti-mouse IgG + IgM (H+L)-HRP	Yes	Jackson lab (115-035-044)
Goat anti-rabbit IgG + IgM (H+L)-HRP	Yes	Bio-Rad (401005)
Goat anti-mouse IgM-HRP	n.d.	Jackson lab (115-035-020)
Goat anti-rabbit IgM-HRP	n.d.	Abcam (Ab97195)
Goat anti-mouse IgG F(ab’)_2_-HRP	n.d.	Jackson lab (115-036-006)
Goat anti-rabbit IgG F(ab’)_2_-HRP	n.d.	SantaCruz (sc-3837)

n.d., not detected

### The Accuracy of the M&R LE Protein Marker

**Fig 5:**
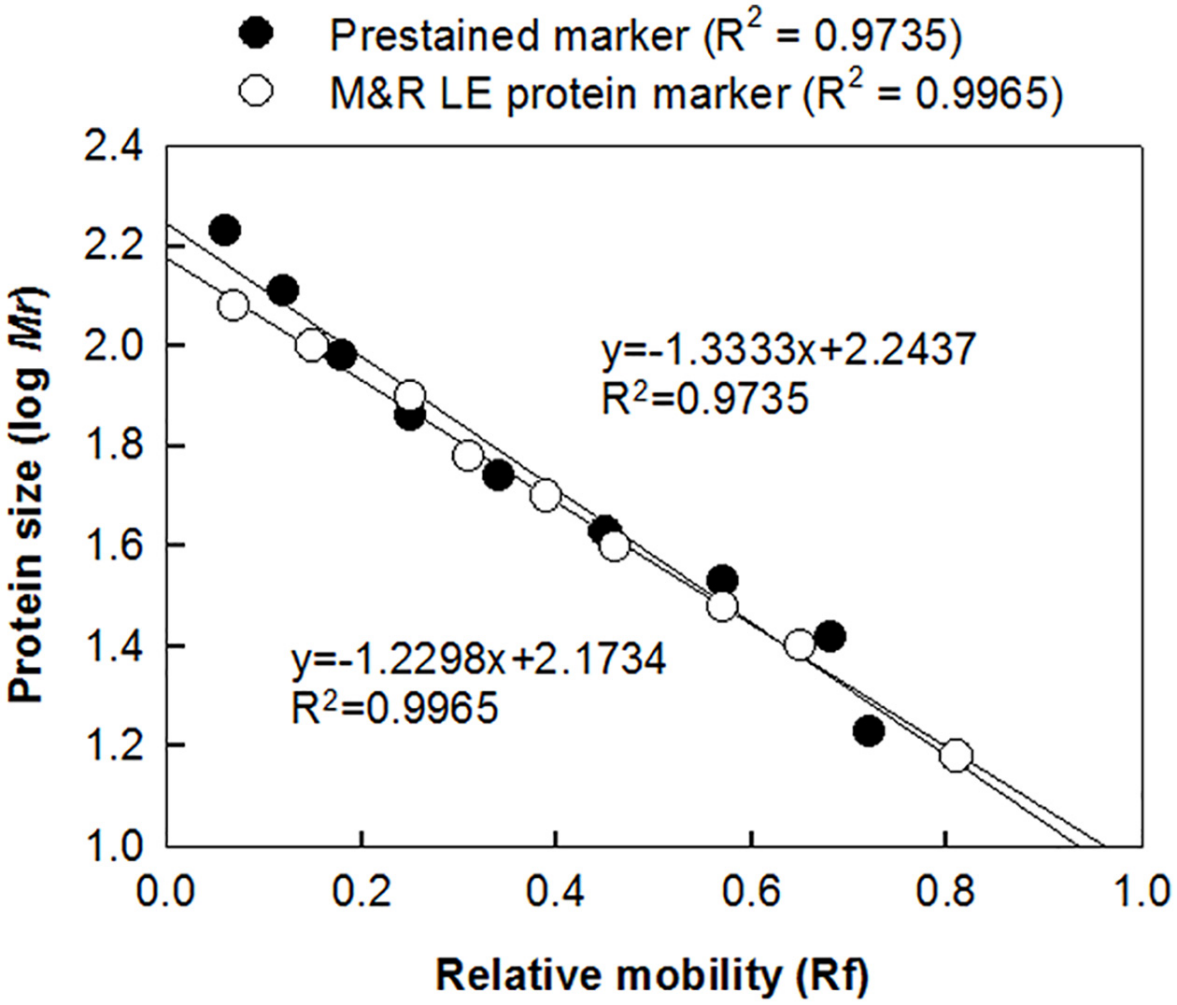
Relationship between molecular weight and electrophoretic mobility of M&R LE protein marker. The relative mobilities of the M&R LE protein marker (○) or commercial prestained markers (●) were plotted against the logarithmic molecular weights of each marker protein. Simple linear regression was used to determine the best fit lines. The R^2^ value were calculated by SigmaPlot 10 software.

The migration of the M&R LE protein markers in immunoblots was analyzed to determine the accuracy of the markers. The relative mobility of the component protein markers, which is defined as the ratio of the protein migration distance to the distance of the running front in the SDS-PAGE, was plotted against the logarithm of the protein molecular weight. Linear regression analysis demonstrated that the relative marker mobilities and the logarithm of the marker molecular weights were inversely correlated (R^2^ = 0.9965), indicating that the M&R LE protein marker is highly accurate (Fig 5). The correlation coefficient of the fitted line for a commercial prestained marker (Fermentas, Catalog No. SM0671) was appreciably lower (R^2^ = 0.9735), possibly owing to altered electrophoretic mobilities caused by dye molecules on the marker proteins. These results indicate that the M&R LE protein marker displays precise molecular weights as compared with a commercial prestained marker.

## Discussion

Direct visualization of dye-free and denaturable molecular weight markers on immunoblots can provide convenient and accurate analysis of protein molecular weights. In this study, we developed a novel M&R LE protein marker which incorporates linear antibody-binding epitopes derived from mouse IgG1 and rabbit IgG Fc regions (M&R LE epitope). The M&R LE protein marker is recognized by HRP-conjugated anti-mouse and anti-rabbit secondary antibodies under denaturing conditions and auto-detects a regular molecular weight ladder on immunoblot films. This novel strategy may provide a more accurate, convenient and effective tool for proteomic research.

Antibodies are widely used in several types of immunoassays such as immunoblotting [18, 19], immunohistochemistry (IHC) [14], immunocytochemistry [20], immunoprecipitation (IP) [21], enzyme-linked immunosorbent assay (ELISA) [22, 23] and fluorescent *in situ* hybridization [24, 25]. Because mice and rabbits are the most commonly used hosts for immunization and primary antibody production [24], commercial secondary antibodies that recognize mouse and rabbit antibodies are widely available [26]. Furthermore, the majority of primary antibodies are immunoglobulin G (IgG) due to the high antigen-binding affinity of IgG antibodies as compared to IgM and IgA antibodies [27]. We therefore identified and fused linear epitopes (M2R2) derived from mouse and rabbit IgG antibody Fc regions as a secondary antibody-detectable tags to generate the M&R LE protein marker. Our results show that the M&R LE protein marker can be recognized by several commercial anti-mouse and anti-rabbit secondary antibodies (Fig 4, Table 2). The M&R LE protein marker should be generally useful for mouse and rabbit antibody based studies. Furthermore, the M&R LE protein marker can be used as a positive control for secondary antibodies against mouse and rabbit IgG Fc.

A protein ladder which can tolerate denaturing conditions is important to the development of molecular weight markers. Previous study had suggested that the linear epitope can successfully bind by antibodies after heating treatment (100°C) [12]. Unlike current auto-detecting protein markers (e.g. GFP- or Protein A/G-based protein ladder) [4, 6, 28], our results show that the M&R LE protein marker can be recognized and auto-detected on film by secondary antibodies under denaturing conditions (boiling and reducing dye treatment). This can be attributed to selection of linear antibody-binding epitopes from the Fc region of mouse and rabbit primary IgG antibodies. The ability of the M&R LE protein marker to function in reducing gels may allow more accurate molecular weight determination on SDS-PAGE and immunoblots.

The M&R LE protein marker can be widely applied in immunoblots that use anti-mouse or anti-rabbit IgG Fc secondary antibodies in immunoblot. However, it cannot be used with assays that employ anti-Fab, anti-IgA or anti-IgM secondary antibodies nor secondary antibodies against other species. In this study, we provide a novel strategy based on the identification of linear epitopes in the primary antibody that can be used as a detectable tag for secondary antibodies. This concept should be extendable to selection of linear epitopes from different portions of antibodies such as the Fab region or to fusing linear epitopes of primary antibody from two or more different species to generate second-generation auto-detected protein markers.

In summary, we developed a novel M&R LE protein marker for protein electrophoresis. It has apparent advantages over traditional protein markers as follows: (1) the marker can be recognized by secondary anti-mouse and anti-rabbit antibodies under reducing conditions, (2) the marker displays precise molecular weights, (3) the marker can be auto-detected on film in immunoblots, (4) it is suitable for any anti-mouse and anti-rabbit IgG Fc secondary antibodies and (5) can act as a positive control for secondary antibodies. We expect that the M&R LE protein marker will provide a denaturable, accurate and convenient standard for protein electrophoresis.

## Supporting Information

S1 Fig The sensitivity of M&R LE protein markers to detection by anti-mouse or anti-rabbit IgG Fc secondary antibodies. Serially-diluted M&R LE protein markers were directly stained by HRP-conjugated (A) anti-mouse or (B) anti-rabbit IgG Fc secondary antibody at concentration of 0.8–1.6 μg/ml. The molecular weights of a commercial pre-stained protein marker (right) and the M&R LE protein markers (left) are indicated.
(TIF)
