# Supplementary figures and images for: A Secondary Antibody-Detecting Molecular Weight Marker with Mouse and Rabbit IgG Fc Linear Epitopes for Western Blot Analysis
Source: PLoS One. 2016 Aug 5;11(8):e0160418. doi: 10.1371/journal.pone.0160418 (PMC4975442; doi:10.1371/journal.pone.0160418)

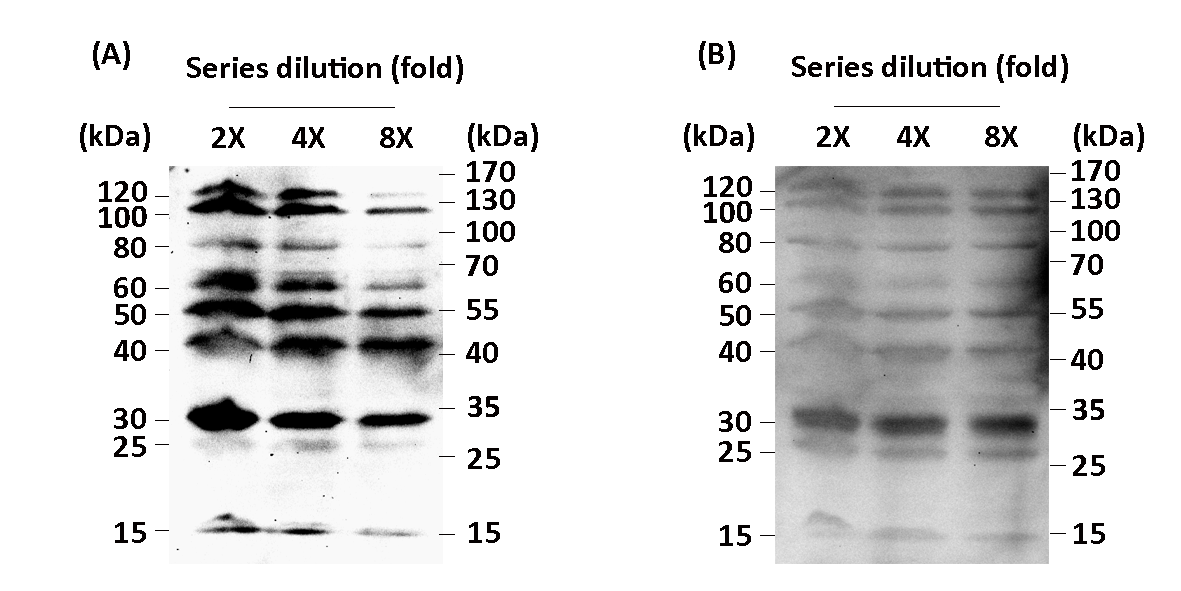

Supplement: S1 Fig — Serially-diluted M&R LE protein markers were directly stained by HRP-conjugated (A) anti-mouse or (B) anti-rabbit IgG Fc secondary antibody at concentration of 0.8–1.6 μg/ml. The molecular weights of a commercial pre-stained protein marker (right) and the M&R LE protein markers (left) are indicated. (TIF) [file pone.0160418.s001.tif]
